# Supplementary material for: The Helicobacter pylori AI-clinician harnesses artificial intelligence to personalise H. pylori treatment recommendations
Source: Nat Commun. 2025 Jul 14;16:6472. doi: 10.1038/s41467-025-61329-5 (PMC12259899; doi:10.1038/s41467-025-61329-5)
Supplement: Supplementary file 1 — Supplementary Information [file 41467_2025_61329_MOESM1_ESM.pdf]

The *Helicobacter pylori* AI-Clinician Harnesses Artificial Intelligence to Personalize *H pylori* Treatment Recommendations

Supplementary Notes.

Bias and Representativeness.

Hp-EuReg is a multi-national, multi-center clinical retrospective spanning Europe. Naturally, the results of analysis of this dataset will be biased to European demographics. However, within these demographics, steps were taken to ensure a balanced representation of sex, age, and race. Of the post-processed dataset, 15,622 (41.1%) individuals were male and 22,406 female (58.9%) with 21 unrecorded sex (<0.001%). As the study recruited across European countries, the majority of patients recruited in the study were Caucasian (n=33,725; 88.6%), with 435 Asian (1.1%), 242 Black (0.6%), and 2699 (7.%) Other, with 948 unrecorded (2.5%). Finally, 9779 (25.7%) were 40 or under, 8008 (21.0%) were between 40 and 50, 8,560 were between 50 and 60 (22.5%), and 11,682 (30.7%) were 60 or older. (For a full list of clinical variables and their relative quantities in the dataset, see *Supplementary Data 1.*)

Performance on Simulated Data

To demonstrate the effectiveness of our model in detecting heterogeneous optimal treatment choices, we generated a simulated dataset of 10,000 patients with 100 binary features. To model an asymmetric dataset (unbalanced classes) 7000 patients were assigned one random combination of binary variables, while another group of 3000 was assigned a different random combination of variables. (Supplemental Fig. 1A) Noise was added to the dataset at various levels by flipping bits (one-hot encoded variables) to obscure the structure of variables determining outcome, similar to real-world datasets. Two simulated treatments were used in the dataset, where treatment A was given 90% success in group A of patients, and treatment B was given 80% success in group A. Likewise, treatment B was given 90% success in group B whereas it was only given 80% in group A.

Data was generated and split into a balanced and randomized 90-10 training-testing distribution. Here we consider a 'correct' optimal drug recommendation to be one where the drug with 90% effectiveness in the patients' group of origin is recommended (For example, drug A to a patient from group A). During the testing phase, Q scores for both treatments (optimal and non-optimal) were examined, demonstrating highly accurate optimal drug recommendations. (Supplemental Fig. 1B) Noise levels of 5, 10, 25, 50, 75, and 99% were tested to evaluate the model's performance in the presence of noisy data. The percentage of patients correctly identified in each noise level was 98.9% (5% noise), 94.2% (10% noise), 92.1% (25% noise), 92.3% (50% noise), 82.4% (75%), 72.0% (99% noise) demonstrating that the model is capable of detecting patient heterogeneity in optimal treatment even in the presence of highly noisy data. (Supplemental Fig. 1C) While higher levels of noise are artificial and used solely as a stress

test, it illustrates the robustness of the model in distinguishing between simulated patient subgroups based on subtle patterns in the data. Even under extreme noise, some residual structure remains, which the model can exploit—despite being difficult for humans to perceive. This parallels real-world clinical settings, where individual variables (e.g., age, sex, comorbidities, concurrent medications) may interact in complex ways that influence treatment success.

A second, more complex simulated analysis was performed for hyperparameter tuning. In this case, 10 unique simulated patient types were generated, with a unique random pattern of 70 one-hot encoded patient variables, for 50,000 simulated patients total. In each simulated patient 'type' a single optimal treatment out of 10 possibilities is given 90% effectiveness, with all other treatments providing 70% effectiveness. An additional 5% noise is added to the data to simulate real world variability. A hyperparameter grid testing multiple combinations of batch size, steps per optimization, deque size, and learning rate is sampled to include every combination, and a model trained using a random sample of 80% of the data. As a metric of success, a score is generated between 0 and 1 which represents the fraction of patients in the testing dataset (remaining 20%) which was correctly assigned the treatment corresponding to 90% success rate in the simulated training group. An optimal combination favoring small batch sizes and a fast-learning rate was found to perform best and used for analysis of real-world data. (For a full description, see *Methods*)

Model Validation

To validate the performance of our model on an external dataset, independent new patient records from the Hp-EuReg dataset from February 14th 2024 to March 19th 2025 (n=7186) were used to examine the distribution of recommended treatments and relative success rate of AI-recommended treatments to those which differed from clinician prescribed treatments. (In terms of sex representation, there are 2894 males and 4292 females.) If we consider all bismuth therapies as a single entity for comparison as we did in the training dataset results, 67.1% of patients are recommended a bismuth therapy (compared to 65.5% in the original dataset), 22.0% were recommended a quadruple non-bismuth therapy with clarithromycin, amoxicillin, and metronidazole (compared to 15.5% in the original dataset) and 10.9% did not receive consistent recommendations above the required threshold (compared to 19.0% in the original paper, suggesting an improved consistency in recommendation on the newer dataset). If we consider different bismuth regimens separately, 58.4% of patients with consistent recommendations were recommended a quadruple non-bismuth therapy with clarithromycin, amoxicillin, and metronidazole (compared to 51.5% in the original dataset), 30.3% were recommended Pylera® (30.4% in the original dataset), and 11.2%% were recommended a quadruple bismuth therapy with clarithromycin, amoxicillin, and bismuth salts (18.1% in the original dataset). Taken together these results demonstrate a very similar ratio of bismuth salt therapies to non-bismuth quadruple therapies.

Overall, the success rate of treatments which agreed with AI recommendations (n=138) was 92.8% (CI: 88.4-97.1%) compared to a success rate of 87.4% (CI: 86.7-88.2%) for treatments which did not agree with AI recommendations (n=7048), resulting in a net improvement of 5.4% (compared to an improvement of 6.0% in the dataset used to train and test the AI Clinician originally). This result suggests a high improvement in treatment successes would have also been made by using the recommendations provided by the AI clinician in the validation set, and therefore applicability of the AI Clinician to new patients.

Comparison to Other Models

We quantify the success of models used in the comparison by comparing the overall success rate of treatments used by clinicians that agreed with model recommendation (whether the model is isDQN, LR, RF, or SVM-based). LR models were designed to take the 10 patient features most correlated to eradication in each treatment. Since RF models are usually not used to predict quality of an assortment of actions, we modified the formulation to eliminate the softmax which would usually be used for label prediction, and directly compared predicted probabilities of the success of certain treatments. SVMs were also coded to return a probability of each result. For each treatment, a separate model (RF, for example) is trained to predict the success of eradication. Then, the probabilities of success for each treatment are compared, and the highest probability is selected as the system's 'recommended treatment'. 100 Iterations of bootstrapping are used for each model to calculate a standard deviation of results. Overall, for LR-based architecture a mean success rate of 88.3% (SD=0.2%) was observed, for RF-based architecture a mean success rate of 88.3% (SD=0.2%) was observed, for SVM-based architecture a mean success rate of 88.3% (SD=0.2%) was observed. In comparison, isDQN modelling saw a success rate of 94.1%, demonstrating the superiority of the approach. Prior antibiotic exposure is a known factor which can reduce the effectiveness of a therapy. Similarly, in order to investigate the potential of patient antibiotic resistance alone to predict optimal treatment, a logistic regression architecture as described above was generate for each treatment using only the one-hot encoded variables referring to antibiotic resistance (included resistance possibilities: clarithromycin, nitroimidazole, quinolone, amoxicillin, tetracycline, no resistance, or no resistance check performed. It is worth noting as a shortcoming this data is only present for 3980 patients in the dataset (10.46% of the dataset). Therefore, we drop treatments where less than 100 patients who received them also had antibiotic resistance information available (13 treatments dropped). Overall, the mean success rate of recommended treatments is 89.1% (SD=0.8%), again compared to an effectiveness of the AI Clinician of 94.1% and demonstrating the ineffectiveness of using simple variables such as antibiotics resistance for optimal treatment recommendation.

Discussion of Model Examination by MI-CLAIM Criteria

To ensure transparent reporting of our AI model, we employed the MI-CLAIM criteria[1], including the MI-CLAIM Checklist (Supplementary Table 2). As we are working with retrospective clinical data, the primary metric to evaluate algorithmic performance is success rate of treatment successes where AI agreed with real-world treatment compared to those in which it did not. Bootstrapping was also performed to generate a 95% confidence interval. In addition, since the accepted clinical guideline for eradication success is a treatment which achieved at least 90% eradication, we use this same success rate to justify the clinical utility of the model – demonstrating a success rate of 94.08% in this dataset (CI: 93.21%, 94.95%). Therefore, our first model examination technique was the predicted eradication success of treatment. Our second examination technique was random forest modelling of subsets of patients recommended the same therapy by AI for extrapolation of feature importances of each variable (full results available in Supplementary Data 1). These results allow for model interpretability at the case level if examination methods are uninterpretable as individual patient variables demonstrated to have a high importance score can be compared to patient variables relevant to the individual (for example: region of residence, race, additional medication intakes) to find which variables belonging to the individual are driving the AI Clinician's decision. Model robustness to shifts in data has been extensively tested via 10-fold cross-validation over 50 repeats, meaning that independent models were trained on diverse sub-samples of input data.

Comparison of Additional Data Splits

As a priority of our reinforcement learning framework was to develop a system which has trained on as large of an input as possible before making recommendations, ten-fold cross validation was chosen to allow for sufficient training examples in heterogeneous populations. However, to test that the performance of the model would not have been similar with smaller splits, we also ran models using five and threefold cross validation for comparison. Overall, using five folds resulted in a success rate of 92.8% for recommendations which agreed with clinician treatment (-1.3% compared to ten folds) and using three folds resulted in a success rate of 85.0% (-9.1% compared to ten folds). Overall, these results demonstrate successive improvements of increased training data in our model.

Expanded Discussion of RF Variable Importance in Patients Recommended the Same Treatment

As discussed in Results, RF models were built to predict the likelihood an individual would be recommended a given treatment by the AI Clinician based on their variables. Interestingly, a patient's region of origin is a highly relevant variable for all treatments. For example, patients recommended Pylera® are more likely to be from a southwest region, which could likely be due to the model observing Pylera® has a higher success rate in these regions compared to eastern regions where it is often unavailable and therefore its effectiveness remains less established. Interestingly, an eastern region is important for being recommended both

non-Pylera® bismuth quadruple and non-bismuth quadruple therapies. This may be partially due to the same reasons described for Pylera®, however other likely driving factors are differences in diet, lifestyle, and the genetic makeup of the bacteria – to name a few. Beyond region, the most consequential patient variables related to treatment recommendation relate to other medications, with acetylsalicylic acid, rebamipid, and probiotics demonstrating particular association to specific treatments. This is likely due to complex interactions between other drugs, the gut microbiome, and individual. Indeed, taking concurrent medication showing up as important in Pylera® and bismuth quadruple therapies, suggesting that a wide variety of complex drug interactions may have a negative impact on treatment effectiveness if not paired with a bismuth salt regimen. A Caucasian ethnicity was important in being recommended non-bismuth quadruple therapies, perhaps suggesting an interplay of genetic factors with treatment efficacy. Finally, the lack of heartburn showing a high correlation to bismuth salts regimens generally could demonstrate that heartburn is a particularly relevant factor in determining whether or not a bismuth salt is needed in a particular patient's regimen.

Examining Sex Differences in Model Performance via Internal Validation

In order to investigate the performance of the AI Clinician on biological males compared to biological females, we carried out a segregated analysis comparing the success rate of AI-recommended therapies versus non AI-recommended therapies with 1000 steps of bootstrapping. In males, we found a success rate of 95.1% (CI: 93.8-96.3%, n=1109) of AI-recommended treatments compared to 89.0% (CI: 88.4-89.4%, n=14,513) whereas in females, we found a success rate of 93.5% (CI: 92.3-94.6%, n=1879) of AI-recommended treatments compared to 87.5% (CI: 87.1-88.0%, n=20,548). Taken together, these results show a highly similar rate of success for men and women in recommendations, within statistical error as evidenced by the confidence intervals. In both cases there is a slightly higher average success rate in both AI-recommended and non AI-recommended therapies in men compared to women.

Response to Sex and Gender Reporting Requirements

In compliance with Nature Communications' guidance on sex and gender reporting and the SAGER guidelines, we provide the following clarifications regarding our study:

1. Applicability of Research Findings by Sex/Gender

The research findings apply to all patients regardless of sex or gender. There is no indication that results are specific to one sex or gender. Therefore, sex-specific findings are not included in the title or abstract.

2b. Studies Involving Human Research Participants

Our study is based on a large real-world clinical registry (Hp-EuReg) involving human participants.

Was sex and/or gender considered in the study design?

Yes, sex was included as a variable in the model and considered during both model development and performance analysis. The dataset includes sex-disaggregated information, and we examined the distribution and influence of sex on treatment recommendations and eradication outcomes.

Was sex/gender determined by self-report or assigned, and how?

Sex was recorded as part of routine clinical documentation at the time of patient intake. While the specific method may vary slightly between centres, sex was typically determined based on clinician assessment or patient self-report. Gender identity was not systematically recorded in the Hp-EuReg dataset and was therefore not included in the analysis.

3. Disaggregation and Reporting of Sex-Based Data

Sex-disaggregated data have been incorporated into our analyses. We conducted subgroup analysis to examine model performance across male and female patients. These findings are included in the Supplementary Materials. As consent for individual-level sex-disaggregated reporting was obtained via the registry's ethical framework, we have included overall distributions in the manuscript and provide sex-disaggregated metrics in the source data where appropriate.

For further information, please see the SAGER Reporting Checklist for Human Studies (*Supplementary Information 1*).

Supplementary Reference

1 Norgeot, B. *et al.* Minimum information about clinical artificial intelligence modeling: the MI-CLAIM checklist. *Nature medicine* **26**, 1320-1324 (2020).

a

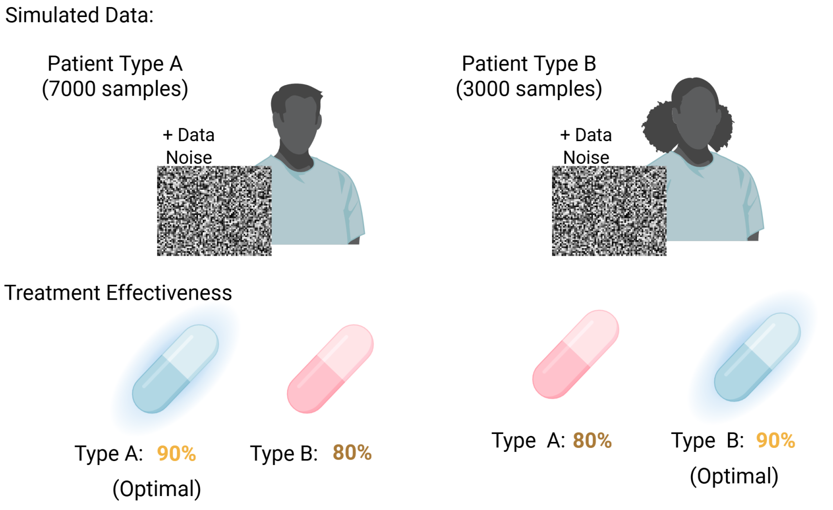

b

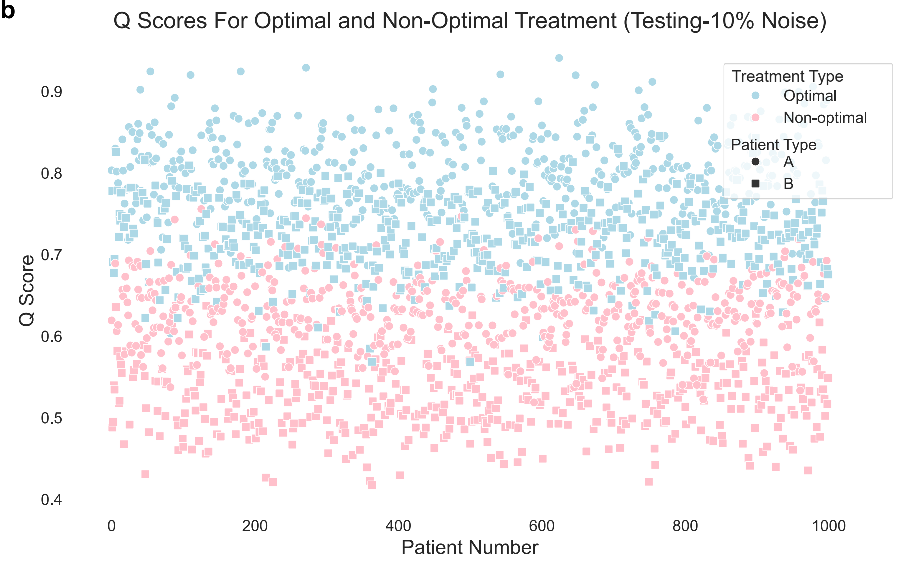

c

Noise (%)	Correct Optimal Recommendations (%)
5.0	98.9
10.0	94.2
25.0	92.1
50.0	92.3
75.0	82.4
99.0	72.0

Supplementary Fig. 1: Performance on Simulated Data. a A theoretical dataset was generated to demonstrate the sensitivity of the AI Clinician in detecting optimal treatment relative to a heterogenous patient population, where drug A is 90% effective in population A and drug B is 80%, and vice versa for population B. The theoretical dataset consisted of n=10000 patients with 100 binary variable features, and distributed into a randomized and balanced 90-10 training-testing split. Reward is assigned as +1 if successfully treated and -1 if unsuccessfully treated. To test the model's performance in the presence of unbalanced classes, 7000 patients of population A are generated and 3000 of populated B. Noise was added to the dataset as random introductions of 0's and 1's at varying levels to simulate increasingly noisy real-world data. **b** Q Scores for recommendations of both the optimal and non-optimal treatment (two per patient) were plotted during the testing phase, with recommendations of the drug optimal for that simulated patient highlighted in green and patient type distinguished by shape. Overall, the AI clinician correctly ascribes optimal treatments for both patient types with higher Q scores than non-optimal, with variation due to noise. **c** Repeating analysis over many different noise levels showed that the majority of patients are correctly recommended the optimal drug even with increasing noise, demonstrating the ability of the model to detect heterogeneity in patient populations even in highly noisy data. Figure 1a Created in BioRender. Higgins, K. (2025) https://BioRender.com/7m14s87.

Supplementary Information 1 SAGER Reporting Checklist for Human Studies

Section	Recommendation	AI clinician work
**Title / Abstract**	If the study findings apply to only one sex or gender, this should be explicitly stated.	*Not applicable* — our study applies to both sexes.
**Introduction**	Mention if sex/gender is expected to influence outcomes or decisions.	Prior studies have suggested potential sex-based differences in antibiotic resistance or treatment response, which may influence personalized therapy for H. pylori.
**Methods – Study Design**	State whether sex/gender was considered in the model and how it was recorded.	Sex was included as an input feature in the reinforcement learning model. It was collected via routine clinical reporting at each site, typically based on self-report.
**Methods – Data Analysis**	Describe how sex/gender was handled: included in model, used in subgroup analysis, etc.	Sex was included in the input vector to enable the model to learn potential interactions between sex and treatment efficacy. Subgroup analysis was performed by stratifying the predictions of the model and comparing success rates respectively. (See Appendix)
**Results**	Report overall sex distribution and, where applicable, disaggregated results.	Of the 38,049 patients in the training dataset, 58.9% were female and 41.1% were male. Sex was among the top 15 features contributing to model decisions (see Supplementary Table 4). In our external 7186 patient validation cohort 59.7% were female and 40.3% were male. When stratifying by sex we found a success rate of AI recommended therapies of 93.5% (CI: 92.3-94.6%) in females and 95.1% (CI: 93.8-96.3%) in males, demonstrating a non-statistically significant difference between sexes. (Full description of results in Appendix)
**Discussion**	Address the role of sex/gender in the model or outcomes. If no effect found, say so.	Although the model incorporated sex as a predictive feature, we did not observe strong performance differences attributable solely to sex.
**Supplement / Data Availability**	Provide sex-disaggregated data where available and ethical.	Descriptions of sex-specific representation in the dataset available in Appendix, Supplemental Table 1.
**Ethics**	Clarify how sex/gender information was collected and governed ethically.	Sex data were collected under the ethical protocols of the Hp-EuReg registry, with informed consent and approval for use in anonymized research.

Supplementary Table 1 Countries to Region Mapping

Region	Country
East	Ukraine
East	Serbia
East	Bulgaria
East	Turkey
East	Russia
East	Romania
East	Albania
East	North Macedonia
East	Bosnia and Herzegovina
East	Kosovo
East	Moldova
East	Montenegro
East-centre	Croatia
East-centre	Poland
East-centre	Hungary
East-centre	Latvia
East-centre	Lithuania
East-centre	Greece
East-centre	Slovenia
East-centre	Czech Rep
East-centre	Azerbaijan
East-centre	Slovakia
East-centre	Malta
East-centre	Armenia
South-west	Portugal
South-west	Spain
West-centre	France
West-centre	Austria
West-centre	Belgium
West-centre	Germany
West-centre	Italy
North	The United Kingdom
North	Finland
North	The Netherlands
North	Ireland
North	Israel
North	Norway
North	Switzerland
North	Sweden
North	Denmark

Supplementary Table 2 MI-CLAIM Checklist

Study Design (Part 1)	Completed: page number		Notes if not completed
The clinical problem in which the model will be employed is clearly detailed in the paper.	☑	3 through 6	
The research question is clearly stated.	☑		1
The characteristics of the cohorts (training and test sets) are detailed in the text.	☑	11 through 15, Supplemental Data 1	
The cohorts (training and test sets) are shown to be representative of real-world clinical settings.	☑	3 through 6	
The state-of-the-art solution used as a baseline for comparison has been identified and detailed.	☑	11, 12, Appendix	

Data and optimization (Parts 2, 3)	Completed: page number		Notes if not completed
The origin of the data is described and the original format is detailed in the paper.	☑	3, 4, 13, Appendix	
Transformations of the data before it is applied to the proposed model are described.	☑	6, 7, 12, 13, 14	
The independence between training and test sets has been proven in the paper.	☑	16, 17	
Details on the models that were evaluated and the code developed to select the best model are provided.	☑	10 through 18, Appendix	
Is the input data type structured or unstructured?	Structured		

Model performance (Part 4)	Completed: page number		Notes if not completed
The primary metric selected to evaluate algorithm performance (e.g., AUC, F-score, etc.), including the justification for selection, has been clearly stated.	☑	1, 13, Appendix	
The primary metric selected to evaluate the clinical utility of the model (e.g., PPV, NNT, etc.), including the justification for selection, has been clearly stated.	☑	1, 6, 7, Appendix	
The performance comparison between baseline and proposed model is presented with the appropriate statistical significance.	☑	1, 6, 7, 8	

Model examination (Part 5)	Completed: page number		Notes if not completed
Examination technique 1[a]	☑	1, 12, Appendix	
Examination technique 2[a]	☑	1, 12, Appendix	
A discussion of the relevance of the examination results with respect to model/algorithm performance is presented.	☑	Appendix	
A discussion of the feasibility and significance of model interpretability at the case level if examination methods are uninterpretable is presented.	☑	Appendix	
A discussion of the reliability and robustness of the model as the underlying data distribution shifts is included.	☑	12, 16, 17, Appendix	

Reproducibility (Part 6): choose appropriate tier of transparency		Notes
Tier 1: complete sharing of the code	☑	Full code available at: https://bitbucket.org/iAnalytica/aiclinician/src/main/
Tier 2: allow a third party to evaluate the code for accuracy/fairness; share the results of this evaluation	☐	
Tier 3: release of a virtual machine (binary) for running the code on new data without sharing its details	☐	
Tier 4: no sharing	☐	

[a]Common examination approaches based on study type: for studies involving exclusively structured data, coefficients and sensitivity analysis are often appropriate; for studies involving unstructured data in the domains of image analysis or natural language processing, saliency maps (or equivalents) and sensitivity analyses are often appropriate.
