# Supplementary material for: The Helicobacter pylori AI-clinician harnesses artificial intelligence to personalise H. pylori treatment recommendations
Source: Nat Commun. 2025 Jul 14;16:6472. doi: 10.1038/s41467-025-61329-5 (PMC12259899; doi:10.1038/s41467-025-61329-5)
Supplement: Supplementary file 2 — Description of Additional Supplementary Files [file 41467_2025_61329_MOESM2_ESM.pdf]

Title: Supplementary Data 1: One-Hot Encoded Patient Feature Summary.

Description: Summary of all patient variables as one-hot encoded for model input. True and false counts represent the number of times a given variable is true or false respectively across all patients. True (vs. false) proportion is calculated as the fraction of true values out of all patients.

Title: Supplementary Data 2: Random Forest Importance Scores.

Description: Importance scores are reported as mean decrease in impurity (MDI) for all patient variables used to predict by random forest modelling whether an individual would be recommended a given therapy by the AI Clinician. Patient variables are ordered by score from highest to lowest for four treatment categories: quadruple bismuth therapy with clarithromycin, amoxicillin, and bismuth salts, Pylera, bismuth therapies generally, or quadruple non-bismuth therapies with clarithromycin, amoxicillin, and metronidazole.
